# Cafe-au-lait spots with resistant hypertension are an indicator of pheochromocytoma: a rare case report

**DOI:** 10.47487/apcyccv.v5i2.351

**Published:** 2024-06-24

**Authors:** Meriam Amri, El Mehdi Tamir, Abdenasser Drighil, Rachida Habbal

**Affiliations:** 1 Cardiology Department, Centre Hospitalo-Universitaire Ibn Rochd of Casablanca, Morocco. Cardiology Department Centre Hospitalo-Universitaire Ibn Rochd of Casablanca Morocco

**Keywords:** Café-au-Lait Spots, Hypertension, Pheochromocytoma, Neurofibromatosis 1, Manchas Café con Leche, Hipertensión, Feocromocitoma, Neurofibromatosis 1

## Abstract

This case report is one of the rare cases of bilateral pheochromocytoma associated with neurofibromatosis type 1. The interest lies in the clinical form in which the diagnosis was revealed. We report the case of a 38-year-old woman admitted for severe hypertension resistant to triple therapy. Clinical examination revealed Cafe-au-lait spots, which are pigmented birthmarks that appear as patches on the skin with a light to dark brown colour. More than six spots are present in an estimated 95% of people diagnosed with neurofibromatosis type 1 (NF1). Abdominal computed tomography (CT) showed bilateral adrenal tumor involvement. The diagnosis of pheochromocytoma was made by measuring urinary Vanillylmandelic acid (VMA). The evolution was favorable after the excision of the tumor, with normalization of blood pressure. In conclusion: resistant hypertension with café au lait spots may indicate pheochromocytoma, especially bilateral, suggesting an underlying genetic condition like NF1, warranting systematic screening.

## RESUMEN

En este reporte se documenta un caso raro de feocromocitoma bilateral asociado a neurofibromatosis tipo 1. El interés radica en la forma clínica en la que se reveló el diagnóstico. Presentamos el caso de una mujer de 38 años que ingresa por hipertensión arterial severa resistente a triple terapia. El examen clínico reveló manchas café con leche, que son marcas de nacimiento pigmentadas que aparecen como manchas en la piel y que son de un color marrón claro a oscuro. Se estima que el 95% de las personas diagnosticadas con neurofibromatosis tipo 1 (NF1) presentan más de seis manchas. La tomografía computarizada (TC) abdominal mostró afectación tumoral suprarrenal bilateral. El diagnóstico de feocromocitoma se realizó mediante la medición del ácido vanilmandélico (VMA) urinario. La evolución fue favorable tras la extirpación del tumor, con normalización de la presión arterial. En conclusión, la hipertensión resistente con manchas café con leche puede indicar feocromocitoma, especialmente si es bilateral, lo que sugiere una afección genética subyacente como la NF1, que justifica un tamizaje sistemático.

## Introduction

Pheochromocytomas are tumours that derive from the neural crest cells and are derived from the chromaffin cells that grow in the adrenal medulla in almost 85% of cases. Most of the time, the tumours are unilateral, and the bilateral presentation is present in only 10% of cases ^1^. This bilateral presentation, in addition to their rarity, makes it a sign of severity because of their genetic background and its relationship with higher cardiovascular risk. The interest in this case of pheochromocytoma lies in its clinical course and diagnosis, which is in association with another dermatological and neuronal tumours like neurofibromatosis type 1 (NF1).

## Case report

We present the case of a female 38-year-old patient. She was diagnosed with arterial hypertension (HT) a year ago and was on triple antihypertensive therapy. She entered the emergency department because of paroxysmal headaches and palpitations. Clinical examination found a conscious, sweaty patient with a hypertensive peak at 229/110 mmHg and tachycardia at 120 beats per minute without any other sign of repercussion, in particular neurological or ophthalmic.

**Figure 1:**
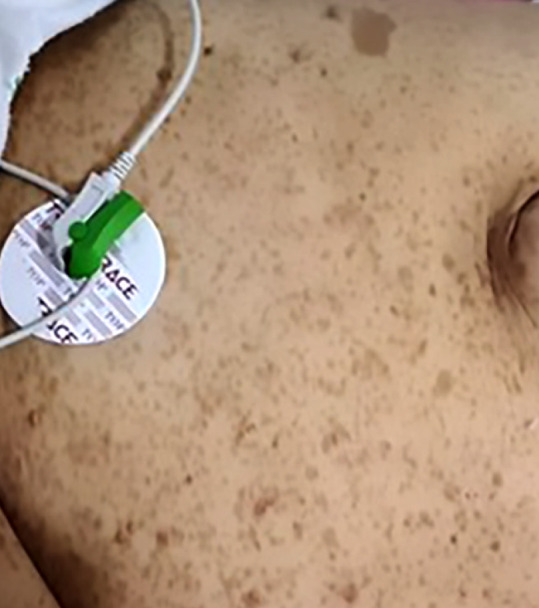
Many brown color lesions mainly located at the trunk.

At physical examination, there was evidence of many brown color lesions mainly located at the trunk (more than six) measuring 16 mm and several neurofibromas on the trunk and limbs (Figure 1). These findings were the pathognomonic criteria that helped to make the diagnosis of neurofibromatosis type 1. The ECG showed a regular sinus rhythm with electric left ventricular hypertrophy. And on echocardiography, there were signs of hypertensive heart disease with symmetrical concentric hypertrophy of the left ventricle. 

There were no abnormal findings at the routine blood tests, and the renal function was also normal. Because of Menard’s triad (headache-palpitations-sweating) in a hypertensive crisis patient, the urinary methoxylated derivatives (Vanillylmandelic acid) were dosed and came out at 40 mg/g of creatinine/24 h (normal value of 2-7 mg/g of creatinine/24 h). Thus, the diagnosis of pheochromocytoma was confirmed.

**Figure 2:**
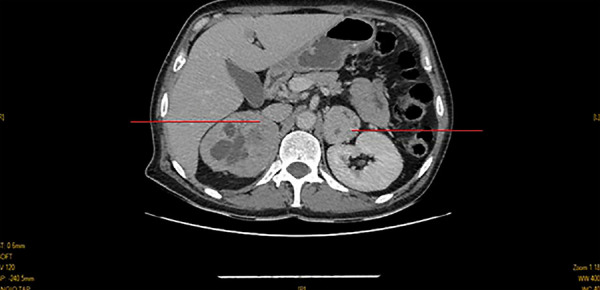
Oval adrenal masses with regular contours (red lines).

Abdominal computed tomography (CT) showed: 2 oval adrenal masses with regular contours measuring 71 x 65.7 mm on the right and 39.5 x 40 mm on the left (Figure 2). The diagnosis imaging was bilateral pheochromocytoma, probably a hereditary form due to its relationship with neurofibromatosis type 1. We managed to stabilize the patient and treat the hypertensive crisis with nicardipine. After that, a bilateral adrenalectomy via laparoscopic surgery was performed. Histological examination confirmed pheochromocytoma without signs of malignancy. The postoperative course was rapid, and the evolution was favorable with normalization of blood pressure and hydrocortisone replacement therapy. We followed the patient regularly for a year without recurrence of the arterial hypertension or any other complication. Unfortunately, we were not able to carry out a genetic test.

## Discussion

HT is the most common chronic disease in adults, with an estimated prevalence of 30% in the general population ^2^. This pathology is the most frequent modifiable risk factor that increase the risk of cardiovascular complications. Although most of the time HT is idiopathic, a 5 to 15% of these patients have secondary hypertension ^3^. If the secondary cause is identified and treated, the hypertension can be solved, and the cardiovascular risk reduced.

Every time we face resistant hypertension, it is necessary to rethink that we haven’t missed a secondary cause, especially pheochromocytoma in particular individuals. Pheochromocytoma is a rare cause of hypertension (0.5 to 1%) but has some characteristic findings in its presentation that we should be aware of ^4^.

Pheochromocytoma is a rare, catecholamine-secreting tumor derived from chromaffin cells. It is most often sporadic and unilateral. However,10% of pheochromocytoma cases can be bilateral ^5^^-^^6^. Nowadays, a genetic test is important in pheochromocytoma diagnosis, as it is most often associated with a hereditary cause, especially in the bilateral form and can help in the prognostic score ^7^. Bilateral pheochromocytomas generally occur in young subjects or in a family setting. It is often associated with other extra-adrenal manifestations, such as multiple endocrine neoplasia type 2, Von Hippel-Lindau syndrome ^8^, or NF 1 ^9^.

Deep interrogation of the family history and physical examination are the first steps to have the suspicion of a hereditary disease. However, family history can be missing if it is a primary mutation ^10^. That is why the international guidelines recommend the systematic realization of a genetic investigation in apparently sporadic pheochromocytomas, especially in cases of young age (59% are hereditary) or in front of multifocal localization (84% are hereditary) ^(^^11^.

The discovery of a hereditary disease is of value to the patient and his family. The risk of recurrence of familial pheochromocytomas is up to 13 times higher than sporadic pheochromocytomas in some series ^12^. therefore, long-term follow-up of all patients with pheochromocytoma is necessary, especially if genetic testing is not available. In our case, we have good results from the clinical examination and follow-up until one year. However, given that we hadn’t done a genetic test, the prognostic and risk of recurrence still need long-term follow-up of the patient and her family ^13^.

Concerning the association of NF1 and pheochromocytoma, it is rare, classically not exceeding 5% of cases ^14^. Neurofibromatosis type 1 is an autosomal dominant disease caused by an NF1 mutation, with a prevalence of 1 in 3000 births. NF 1 has a propensity to develop endocrinological tumors such as pheochromocytoma, which occurs in 0.1-5.7% of cases ^15^. However, according to new studies, pheochromocytoma can be present in up to 4.6% of patients with NF1 ^16^. The classical prevalence of 1-5% seems to be underestimated since there are asymptomatic or non-secreting forms that can have a sudden onset of symptoms. Nowadays, prospective studies with consecutive inclusion reveal a prevalence of up to 8-15% in NF1 ^(^^17^.

Pheochromocytomas in NF1 are 80% asymptomatic and more than 50% non-secreting. When they are secreting, they are often asymptomatic, but they can become symptomatic on the occasion of an event of stress such as a surgical procedure. Given the high frequency of surgical interventions for patients with NF1, this emphasizes the importance of screening for pheochromocytoma in the NF1 population around 35 to 40 years of age, especially before situations that could trigger a crisis, such as an interventional surgery or pregnancy, labor, and delivery ^18^.

This is why systematic screening is recommended in NF1 patients ^19^. The literature recommends screening every five years since the patient is thirty-five years old ^20^. The screening consists of the measurement of metanephrines and the performance of an adrenal scan or MRI. The interest of screening would be to reduce morbidity and mortality through early detection and treatment of the disease and, thus, avoid the severe risks inherent to an unrecognized pheochromocytoma, mainly the cardiovascular risk. 

Bilateral adrenalectomy is the gold standard treatment for bilateral pheochromocytoma. However, this surgical treatment remains controversial due to the need for lifelong corticosteroid therapy and the risk of Addisonian crises associated with bilateral total adrenalectomy ^21^. In fact, up to 23% of patients undergoing bilateral open adrenalectomy for bilateral pheochromocytoma may face the challenge of lifelong corticosteroid dependency, heightening the concern surrounding this approach ^22^. In our case, the evolution was favorable under treatment replacement by hydrocortisone; however, the patient requires close and long-term monitoring to avoid these complications.

Recent advancements have introduced cortical-sparing adrenalectomy, focusing on preserving adrenal function by excising the smaller tumour, thus promoting steroid independence and reducing hypo adrenal crisis risk. This approach is supported by the adrenal gland’s segmental arterial anatomy and dual venous drainage, proving effective through open or laparoscopic techniques. This is particularly crucial for patients with uncertain medication compliance or a limited understanding of Addisonian crisis risks. Nevertheless, cortical-sparing surgery may not be feasible for tumours exceeding 6 cm due to the absence of normal adrenal tissue, as seen in our case ^23^.

In conclusion, In front of resistant arterial hypertension, particularly associated with café-au-lait spots, pheochromocytoma is a diagnosis to discuss. If the pheochromocytoma is bilateral, a genetic pathology is often associated with it and can reveal its hereditary form. Finally, screening for pheochromocytoma should be systematic in the presence of NF1 because of the higher cardiovascular risk in these patients.

## References

[1] Conzo G, Pasquali D, Colantuoni V, Circelli L, Tartaglia E, Gambardella C (2014). Current concepts of pheochromocytoma. Int J Surg.

[2] Mills KT, Stefanescu A, He J (2020). The global epidemiology of hypertension. Nat Rev Nephrol.

[3] Siddiqui N, Daya R, Seedat F, Bulbulia S, Bayat Z (2021). Secondary hypertension An update on the diagnosis and localisation of a pheochromocytoma or paraganglioma. S Afr Fam Pract (2004).

[4] Eya C, Lamia BH, Samira A, Zouleikha K, Imen B, Chekib K (2013). Le phéochromocytome surrénalien bilatéral à propos d'un cas. Pan Afr Med J.

[5] Brunaud L, Ayav A, Bresler L, Klein M, Boissel P (2005). Les problèmes diagnostiques du phéochromocytome. Ann Chir.

[6] Raby L (2011). Bilateral pheochromocytomas incidental finding in a trauma patient. J Emerg Nurs.

[7] Garcia-Carbonero R, Matute Teresa F, Mercader-Cidoncha E, Mitjavila-Casanovas M, Robledo M, Tena I (2021). Multidisciplinary practice guidelines for the diagnosis, genetic counseling and treatment of pheochromocytomas and paragangliomas. Clin Transl Oncol.

[8] Benachour L, Haddam AEM, Meskine D (2016). Un cas de phéochromocytome bilatéral avec maladie de VHL. Ann Endocrinol (Paris).

[9] Plouin PF, Gimenez-Roqueplo AP, La Batide Alanore A, Salenave S, Duclos JM (2000). Progrès récents dans le diagnostic, l'évaluation pronostique et le traitement des phéochromocytomes. Rev Med Interne.

[10] Pacak K, Eisenhofer G, Ahlman H, Bornstein SR, Gimenez-Roqueplo AP, Grossman AB (2007). International Symposium on Pheochromocytoma Pheochromocytoma: recommendations for clinical practice from the First International Symposium. Nat Clin Pract Endocrinol Metab.

[11] Eya C, Lamia BH, Samira A, Zouleikha K, Imen B, Chekib K (2013). Le phéochromocytome surrénalien bilatéral à propos d'un cas. Pan Afr Med J.

[12] Plouin PF, Gimenez-Roqueplo AP, La Batide Alanore A, Salenave S, Duclos JM (2000). Progrès récents dans le diagnostic, l'évaluation pronostique et le traitement des phéochromocytomes. Rev Med Interne.

[13] Alface MM, Moniz P, Jesus S, Fonseca C (2015). Pheochromocytoma clinical review based on a rare case in adolescence. BMJ Case Rep.

[14] Abid S, Rojbi I, Khelifi D, Ben Nacef I, Lakhoua Y, Mchirgui N (2020). Un phéochromocytome dans le cadre d'une Neurofibromatose type 1 révélant un syndrome de NOONAN à propos d'un cas. Ann Endocrinol (Paris).

[15] Képénékian L, Mognetti T, Lifante JC, Giraudet AL, Houzard C, Pinson S (2016). Interest of systematic screening of pheochromocytoma in patients with neurofibromatosis type 1. Eur J Endocrinol.

[16] Zinnamosca L, Petramala L, Cotesta D, Marinelli C, Schina M, Cianci R (2011). Neurofibromatosis type 1 (NF1) and pheochromocytoma prevalence, clinical and cardiovascular aspects. Arch Dermatol Res.

[17] Boudjemaa A, Mezoued M, Bessaid K, Azzouz M (2021). Phéochromocytome asymptomatique au cours d'une NeuroFibromatose de type 1 à propos d'un cas. Ann Endocrinol (Paris).

[18] BeRebai S, Debbabi W, Kammoun F, Chermit S, Marzouk H, Kharrat I (2021). Neurofibromatose découverte en post opératoire suite à une rupture spontanée d'un phéochromocytome méconnu. Ann Endocrinol (Paris).

[19] Belhimer F, Fedala S (2018). Maladie de Von Reklinhausen et phéochromocytome. Ann Endocrinol (Paris).

[20] Képénékian L, Mognetti T, Lifante JC, Giraud AL, Houzard C, Pinson S (2016). Interest of systematic screening of pheochromocytoma in patients with neurofibromatosis type 1. Eur J Endocrinol.

[21] Neumann HPH, Tsoy U, Bancos I, Amodru V, Walz MK, Tirosh A (2019). International Bilateral-Pheochromocytoma-Registry Group Comparison of Pheochromocytoma-Specific Morbidity and Mortality Among Adults with Bilateral Pheochromocytomas Undergoing Total Adrenalectomy vs Cortical-Sparing Adrenalectomy. JAMA Netw Open.

[22] Boaz RJ, Ramakant P, Ebenazer A, Pai R, Rajaratnam S, Abraham D (2011). Role of cortical sparing adrenalectomy and novel variant of mutation in patient with von Hippel-Lindau disease. Indian J Endocrinol Metab.

[23] Alesina PF, Knyazeva P, Hinrichs J, Walz MK (2022). Tailored Approach in Adrenal Surgery Retroperitoneoscopic Partial Adrenalectomy. Front Endocrinol (Lausanne).

